# Au_55_, a stable glassy cluster: results of ab initio calculations

**DOI:** 10.3762/bjnano.8.222

**Published:** 2017-10-25

**Authors:** Dieter Vollath, David Holec, Franz Dieter Fischer

**Affiliations:** 1 NanoConsulting, Primelweg 3, D-76297 Stutensee, Germany; 2 Department of Physical Metallurgy and Materials Testing, Montanuniversität Leoben, A-8700 Leoben, Austria; 3 Institute of Mechanics, Montanuniversität Leoben, A-8700 Leoben, Austria

**Keywords:** amorphous, Au_55_ cluster, glass structure, phase transformation, surface energy

## Abstract

Structure and properties of small nanoparticles are still under discussion. Moreover, some thermodynamic properties and the structural behavior still remain partially unknown. One of the best investigated nanoparticles is the Au_55_ cluster, which has been analyzed experimentally and theoretically. However, up to now, the results of these studies are still inconsistent. Consequently, we have carried out the present ab initio study of the Au_55_ cluster, using up-to-date computational concepts, in order to clarify these issues. Our calculations have confirmed the experimental result that the thermodynamically most stable structure is not crystalline, but it is glassy. The non-crystalline structure of this cluster was validated by comparison of the coordination numbers with those of a crystalline cluster. It was found that, in contrast to bulk materials, glass formation is connected to an energy release that is close to the melting enthalpy of bulk gold. Additionally, the surface energy of this cluster was calculated using two different theoretical approaches resulting in values close to the surface energy for bulk gold. It shall be emphasized that it is now possible to give a confidence interval for the value of the surface energy.

## Introduction

There is a lot of discussion about the structure and surface energy of small nanoparticles. However, this discussion is rather limited due to the fact that there are only sparse experimental results. The Au_55_ cluster is an exception, since this cluster, consisting of a magic number of atoms, is very stable. Consequently, some experimental data are available for comparison with predictions obtained by ab initio calculations. There are a few “magic” numbers indicating hypothetical closed-shell structures. The smallest one of these numbers is 13, with 12 atoms surrounding one central atom. The next two clusters with 55 or 147 atoms add one or two additional shells, respectively. In the outer shell, these clusters contain 42 atoms or 147 atoms, respectively. In an ideal case, these clusters form Mackay icosahedrons, three-dimensional bodies with 12 vertices, the shape of which minimizes the total energy. These clusters may be crystalline or non-crystalline. The latter ones may be amorphous, liquid-like or glassy. The glassy structure is, in comparison with the liquid-like structure, characterized by a reduced heat capacity. In cases where other authors are not differentiating between amorphous (liquid-like) and glassy, the term non-crystalline will be used throughout the rest of this paper.

Since the groundbreaking experimental work of Schmid’s group [1–6], the Au_55_ cluster has been used as model for a small cluster with a magic number of atoms. Their experimental work, and in particular the structural analysis, were possible only by attaching organic ligands to the cluster surface, in order to prevent the clusters from coagulating immediately to larger particles [3]. Without any doubt, the thus obtained structural details do, to some extent, depend on the type of the ligands [4]; however, the structure is not influenced fundamentally. An important result of Schmid’s work is the description of the structure of Au_55_ as a sequence of two shells [1,4]. On average, these clusters are composed of 13 atoms in the center and 42 atoms in the outer shell. From the atoms in the outer shell, 18 atoms are bond to the ligands [1]. Furthermore, these shells show a very broad scattering of the coordination numbers [4]. This fact was taken as an indication for a non-crystalline solid. Additionally, it was found that the bonding energy of these non-crystalline clusters is approximately 20% higher than that of bulk gold [3]. These structural details were confirmed by Vogel and co-workers [7]. Both groups, Schmid et al. [1–6] and Vogel et al. [7], described the shape of the Au_55_ cluster as cuboctahedral. It must be mentioned that there are also findings, especially related to the coordination numbers, that the Au_55_ cluster is possibly structurally close to the face-centered cubic (fcc) structure [8–10]. Baletto and Ferrando prefer the term “low-symmetry structure” instead of non-crystalline in their review article [11]. Further experimental results going far beyond this short introduction, are summarized in a review by Schmid [6]. In addition to these reports based primarily on X-ray diffraction and EXAFS measurements, studies using high-resolution electron microscopy [12–14] were performed. Importantly, these studies pointed out that the structure of small gold clusters was unstable; rather fluctuations between different shapes, primarily, between icosahedral and cuboctahedral habitus were observed [12]. A theoretical description of these fluctuations was given by Sawada and Sugano [15]. However, these authors indicated that the appearance of the cuboctahedral structure has a low probability at room temperature. Based on the experimental studies it can be concluded that an icosahedrally shaped cluster was never observed and, astonishingly, even when these clusters are not crystalline, they have a higher bonding energy than bulk gold.

Having this broad experimental background in mind, it is not surprising that a series of authors tried to describe the structure and the properties of Au_55_ clusters theoretically by means of molecular dynamics or ab initio modeling. These studies resulted in puzzling and often contradictory conclusions regarding the nanoparticle structure: crystalline vs non-crystalline, icosahedral vs cuboctahedral or even unspecific shapes. Doye and Wales [16] described their resulting Au_55_ cluster with the lowest energy as non-crystalline and the structure of the neighboring Au_56_ cluster as fcc-based structure. Cox et al. [17] interpreted their results as highly symmetric. In contrast, Michaelian et al. [18] obtained a low-symmetry structure. Erkoc [19] analyzed a series of gold clusters in the range from Au_13_ to Au_55_ concluding that the stability of these clusters increases with increasing similarity to the fcc structure. Similar results were obtained by Yildirim and Guvenc [20]; these authors found that there is a series of isomers with small differences in the energy. For the central atom of an icosahedron they found a coordination number close to the ideal value of 12. A puzzling result was obtained by Darby et al. [21] as they concluded the Au_55_ cluster had a non-crystalline structure, whereas the neighboring clusters Au_54_ and Au_56_ showed an fcc-based structure. A comparable result for the Au_55_ cluster was discussed by Li and co-workers [22].

This is not really surprising as the energy differences between these different shapes are quite small [20]. Therefore, the results may depend strongly on the starting assumptions for the calculations. Consequently, Soler et al. [23] concluded that it is quite difficult to define a crystalline or a non-crystalline state in case of small metal clusters. Certainly, one must not use these terms as in the case of bulk materials. On one hand, even a “well-crystallized” small cluster will not show symmetries or long-range order as they are common in the bulk. On the other hand, a non-crystalline cluster will certainly show some short-range order. Due to the limited size of the clusters, coordination numbers or the pair correlation function are not unequivocal characteristics of the state of the order. Nonetheless, these quantities are giving possibly the deepest insight to and the best characterization of the crystalline state of a cluster.

Considering this lack of a clear definition, one has to interpret the different results cum grano salis. Moreover, the long list of somewhat contradicting, and to some extent dissatisfying, results on the structure of the Au_55_ nanocluster highlights the necessity to look again at this problem using up-to-date theoretical methods. Therefore, quantum-mechanical calculations within the density functional theory (DFT) framework as implemented in the Vienna ab initio simulation package (VASP) [24] were employed for the present study. This computational process allows one to obtain detailed results with respect to the thermodynamic properties with quantum-mechanical accuracy. Details of the calculations have been published elsewhere [25].

Finally, one may ask which structure results from classical thermodynamic considerations. Based on the thermodynamic data of bulk materials, Vollath and Fischer [26] concluded that particles with a diameter of less than approx. 1.7 nm, which is equivalent to a cluster consisting of approx. 150 atoms, should not be crystalline. This result is, as shown above, well confirmed by the experimental results.

## Results and Discussion

### Basic assumptions and structure of the Au_55_ cluster

As an initial configuration for our calculations, two different arrangements of the gold atoms were selected: (i) a crystalline cluster and (ii) a random arrangement.

The random arrangement is characterized by 55 gold atoms placed in a sphere with a diameter of 1.4 nm. The density of this arrangement (1.25 × 10^4^ kg·m^−3^) is significantly less than the one of liquid gold (1.732 × 10^4^ kg·m^−3^). However, the cluster was allowed to fully structurally relax, hence to adopt also its optimum mass density. The crystalline cluster was first “annealed” at 900 K, “quenched” to 0 K, and finally fully relaxed at 0 K to get rid of any residual forces acting on individual atoms [25].

**Figure 1:**
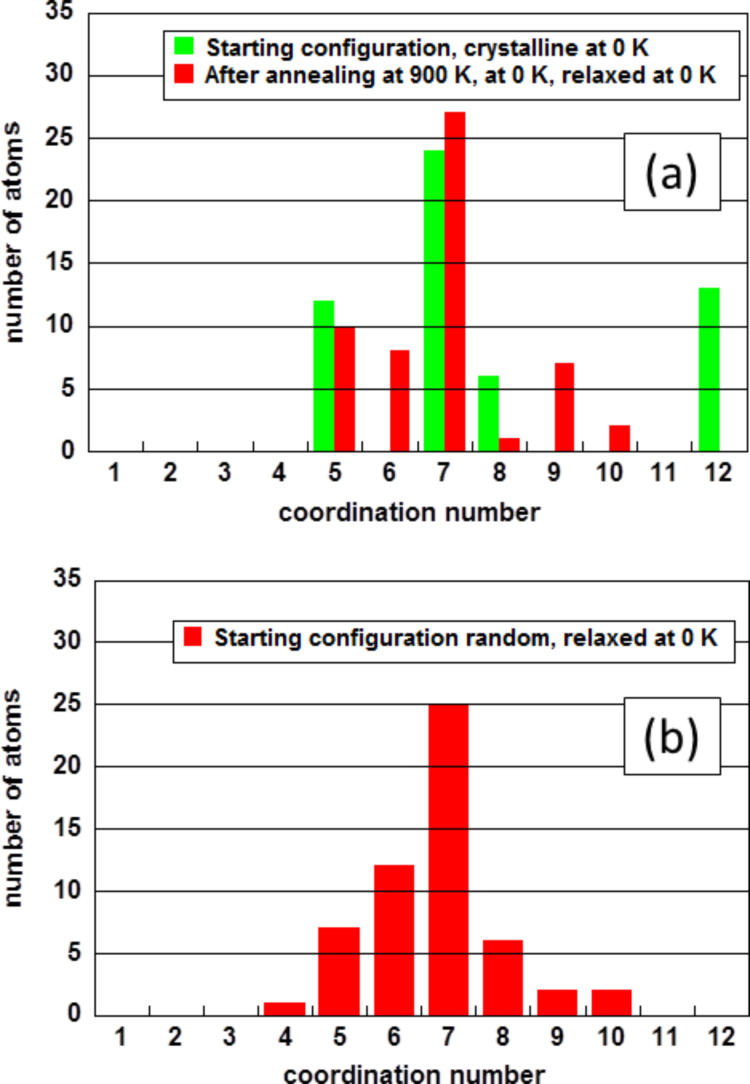
Histograms of the coordination numbers for the two starting configurations crystalline and random. (a) Coordination numbers of the initial crystalline cluster and after annealing at 900 K and relaxation at 0 K. (b) Coordination numbers of the originally random arrangement after relaxation at 0 K.

The resulting arrangements of the atoms were characterized by the histograms of the coordination numbers (Figure 1). In case of the crystalline starting configuration (Figure 1a), the initial coordination numbers (equivalent to the crystalline cluster being only relaxed at 0 K without the annealing procedure) and the coordination numbers after annealing at 900 K and subsequent relaxation at 0 K are displayed. For the random starting arrangement, the histogram of the coordination numbers after relaxation at 0 K is shown in Figure 1b.

The coordination numbers of the crystalline cluster displayed in Figure 1a show that 13 atoms assume the maximum coordination number of twelve as expected for the fcc structure. The most frequent coordination number is seven. However, after annealing at 900 K and relaxation at 0 K, the highest coordination number is reduced from twelve to ten, which is close to 10.9 exhibited by liquid gold [27]. The cluster did not crystallize again after quenching it to 0 K but instead it became amorphous or glassy. The histogram of the random arrangement after relaxation at 0 K in Figure 1b shows a maximum coordination number of ten; the most frequent coordination number is seven. These are the most important parameters to characterize non-crystalline clusters. Besides these two characteristics, the two distributions of the coordination numbers show only differences, as may be expected for non-crystalline particles consisting of a small number of atoms.

### Evolution of the structure

The different behavior of the two starting conditions can be rationalized considering the total energy of the relaxed clusters at 0 K given in Table 1. It clearly shows that the relaxed random starting arrangement yields lower energy than the crystalline one. Furthermore, it is interesting to see that the total energy of the two non-crystalline clusters differ by only about 0.8%.

**Table 1:** Total energy of Au_55_ clusters after relaxation at 0 K.

type of cluster	total energy *E*_particle_ [J·mol^−1^]

crystalline, relaxed at 0 K	−3.46016 × 10^5^
crystalline, annealed at 900 K, relaxed at 0 K	−3.50395 × 10^5^
random, relaxed at 0 K	−3.49365 × 10^5^

**Figure 2:**
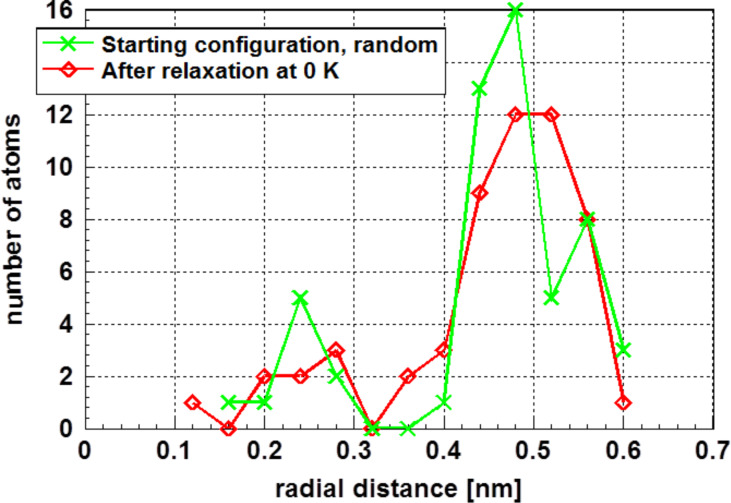
Radial distribution function of gold atoms in the amorphous cluster determined by averaging the number of atoms along a distance (bin) of 2 × 10^−2^ nm. The random starting situation and the annealed crystalline structure, both relaxed at 0 K, are shown. The relaxed cluster is composed of two layers around a central atom.

To describe the structure of these amorphous clusters, the radial distribution function is an important tool. Figure 2 displays such a graph for the random starting situation after relaxation at 0 K. The values displayed in this graph were determined by averaging over a distance (bin) of 2 × 10^−1^ nm. This graph suggests that the relaxed cluster is composed of a central atom and two concentric layers. This tendency and the total energy values are well in line with the experimental results [1–6]. However, one has to keep in mind that also the starting configuration is close to a two-layer system.

**Figure 3:**
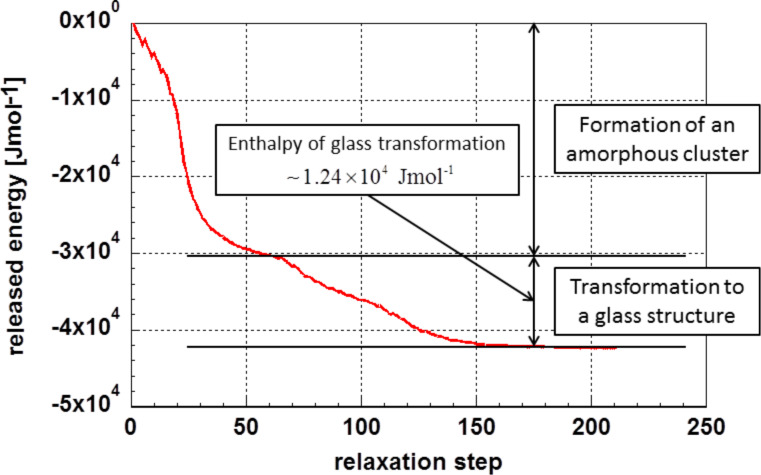
Energy release during relaxation of a random ensemble forming a cluster as function of the relaxation steps.

Figure 3 displays the total energy decrease during relaxation of the initially random cluster at 0 K. One realizes that the energy is released in two regimes. The first regime ranges from relaxation step 1 up to approximately step 65; the second regime starts approximately at step 65 and ends at a relaxation step around 200, which is the final relaxed structure. The second regime describes the formation of the cluster. The energy released during this process is close to the enthalpy of melting of gold, 1.24 × 10^4^ J·mol^−1^ [28].

The two regimes can be explained as: (i) formation of an amorphous cluster from the random arrangement, and (ii) transformation of the amorphous cluster possibly to a glassy one. In the first regime (relaxation steps 1 to ca. 65), one expects the heat of evaporation to be released. This is not the case, since the 55 atoms of the starting configuration were placed within a relatively small sphere with a diameter of 1.4 nm but not in an infinite space.

Obviously, the formation of the glassy cluster in the second regime is a process similar to crystallization as it is connected to a release of energy. This is insofar astonishing as in bulk materials glass formation is not connected to any energy release; only the heat capacity is altered. It may be speculated that this process is responsible for the high stability of “nanoglasses” according to Gleiter [29]. The above analysis leads to the conclusion that the most stable configuration is not liquid-like, amorphous, but instead it is glassy.

**Figure 4:**
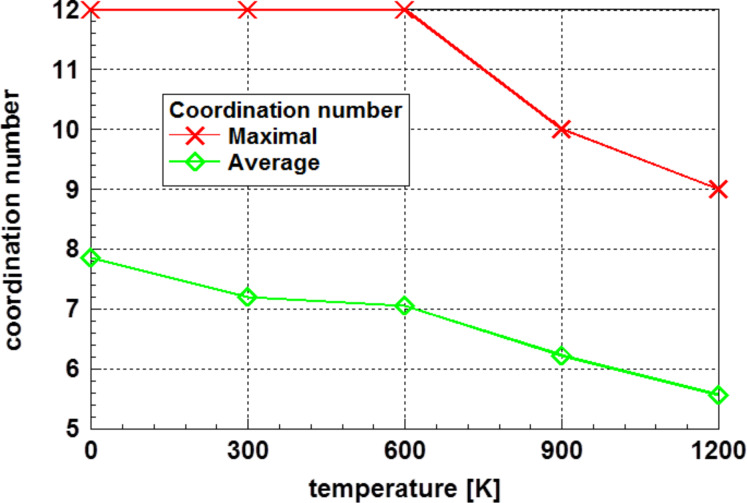
Development of the maximum and the average coordination number of the originally crystalline cluster as a function of the annealing temperature. The reduction of the highest coordination number to values below twelve indicates a complete loss of crystallinity.

The relaxed crystalline cluster was annealed on a series of temperatures up to 1200 K, after which each nanocluster was analyzed for the coordination numbers and energies. Figure 4 displays the development of the maximum and the average coordination number as a function of the annealing temperature. These data suggest a phase transformation in the temperature range around 600 K, which is the highest temperature at which the coordination number of twelve is observed.

**Figure 5:**
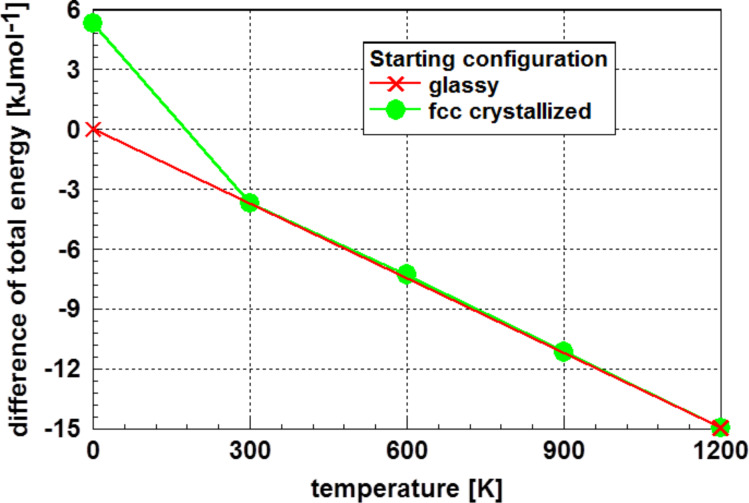
Total energy of the originally crystalline Au_55_ cluster as a function of the temperature, relative to the glassy state at 0 K. It is obvious, the phase transformation of the non-equilibrium originally fcc crystalline cluster to the stable glassy one occurs in the temperature range between 0 and 300 K.

In thermodynamics of bulk materials, a discontinuity of the free enthalpy as a function of the temperature is a typical marker for a phase transformation. Therefore, the total energy of the glassy phase at 0 K was set to zero and the relative values of the total energy of the crystalline cluster at 0 K as well as finite temperatures during its annealing were determined. The results of this evaluation are presented in Figure 5.

Analyzing Figure 5 one realizes a strictly linear relationship of the difference to total energy of the glassy phase at 0 K to the total energy of the annealed fcc cluster as function of the temperature. Extrapolating this linear trend to 0 K yields to a value very close to that of the glassy state. The deviations from the linear function are less than 1%. Consequently, the cluster with fcc structure shows a phase transformation from the non-equilibrium crystalline state to the glassy structure at temperatures below 300 K. Above this temperature, the values of the energy difference correspond to the glassy structure. This phase transformation is also indicated in Figure 4, where the average coordination number is reduced in the same temperature region.

Another quantity characterizing a glassy or an amorphous phase is the average nearest-neighbor distance. It was determined by analyzing the distribution of interatomic distances leading to *d*_Au55_ = (2.804 ± 0.168) × 10^−10^ m. This value is nearly identical to *d*_Au55_ = (2.80 ± 0.01) × 10^−10^ m, a value determined experimentally by Marcus et al. [10] using ligand-stabilized gold clusters. Both values are significantly smaller than the value for bulk gold *d*_bulk_ = 2.855 × 10^−10^ m [30].

### Estimation of the surface energy

#### Energetic conditions

The most straightforward approach to estimate the surface energy of the nanoparticle is to evaluate it as the difference between the total energy of a particle *E*_particle_ and the cohesive energy of the same number *n* of atoms in the bulk *n*·ε_bulk_. This approach was introduced by Medasani et al. [30] to calculate the surface energy of silver clusters using DFT calculations. In their study, the surface energy γ was defined as

[1]
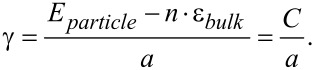


The quantity *a* represents the surface area of the cluster. At the first view, this approach seems logical and simple enough. Hence, it was used by other authors, too [25,31]. However, a deeper thought makes is somewhat questionable, especially for very small clusters. In many cases, small clusters crystallize in a different structure than the bulk material. Small clusters often crystallize in a more symmetrical structure as compared with the bulk material [32] or, contrarily, they are not crystalline at all. This objection is probably not that severe since the energy differences may be small compared to the binding energy. More important is the fact that the binding energy in small clusters may be size-dependent [33]. Furthermore, one can think about magnetic materials in which, generally, the magnetic properties of a surface layer are different to the core of the cluster, or where in contrast to the corresponding bulk material small clusters do not exhibit ferromagnetism.

When applying Equation 1 to the Au_55_ cluster, the total energy of the cluster is *E*_particle_ = −1.927 × 10^7^ J·mol^−1^ (see Table 1) while the cohesive energy is ε_bulk_ = −4.241 × 10^5^ J·mol^−1^ [25]. Nevertheless, one needs also the area of the cluster surface, a problem discussed extensively in [25] and revisited separately later.

Considering the objections explained above, it is desirable to look for a different approach to estimate the surface energy. In this context, the application of the Kelvin equation seems promising, as this equation is well established, e.g., in the analysis of sintering processes. Furthermore, there are many examples in the literature where this equation was successfully applied at the nanometer scale [34–36] and in systems with molecular dimensions [37].

A very consistent derivation of the Kelvin equation was given by Elliot [38] leading to

[2]
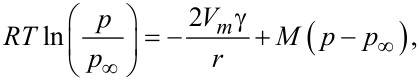


where *R* stands for the gas constant, *p* = *p*_0_·exp(−*Q*/*RT*) is the vapor pressure of the cluster, and *p*_∞_ = *p*_0,∞_·exp(−*Q*_∞_/*RT*) is the vapor pressure of a flat plane. *Q* and *Q*_∞_ are the enthalpies of sublimation of the cluster and of a flat plane, respectively. The quantity *V*_m_ stands for the molar volume of the cluster, γ for the surface energy, and *r* for the radius of the cluster. As the ab initio calculations were performed at *T* = 0 K, the limit at this temperature had to be calculated as

[3]
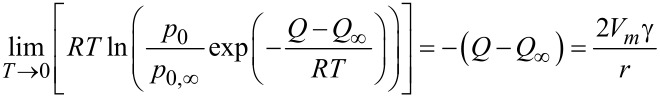


yielding for the surface energy γ at *T* = 0 K:

[4]
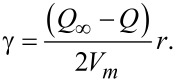


If a spherical geometry is assumed, using *V_m_* = *N*_A_·ν/*n* = *N*_A_·*ar*/3*n,* where ν stands for the volume and *n* for the number of atoms of the particle, Equation 4 becomes

[5]
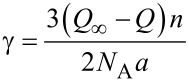


with *N*_A_ being the Avogadro constant. Equation 5 contains, similar to Equation 1, only the surface area per cluster as geometrical input. This fact simplifies the comparison of the results of these two approaches for calculating the surface energy.

The enthalpy of sublimation of a flat surface is *Q*_∞_ = 3.684 × 10^5^ J·mol^−1^ [30]. Nanda [39] applied Equation 4 at temperatures *T* > 0 by assuming *p*_0_ = *p*_0,∞_; however, without justifying it. Possibly, as a consequence of this assumption, Nanda et al. [40–41] obtained astonishingly high values for the surface energy of gold and silver using Equation 4.

**Figure 6:**
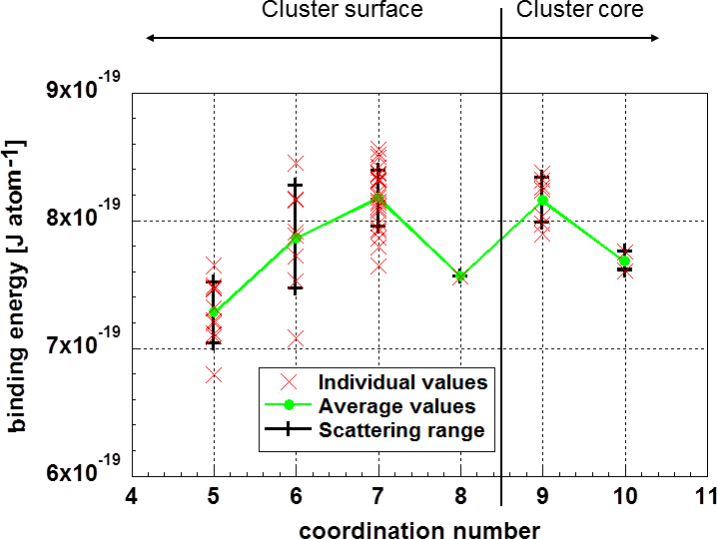
Energy necessary to remove one atom from an Au_55_ cluster, which is equivalent to the enthalpy of sublimation. Coordination numbers of eight or less are found at the surface; higher coordination numbers are in the cluster core. The data correspond to the glassy Au_55_ cluster (initially crystalline structure annealed at 900 K and subsequently fully relaxed at 0 K).

The enthalpy of sublimation, *Q*, for the Au_55_ cluster can be calculated by determining the energy necessary to remove one atom from the cluster, i.e., the binding energy of one atom. These data as function of the coordination number are depicted in Figure 6 for the glassy cluster (crystalline initial structure annealed at 900 K and relaxed at 0 K).

At this point, the question arises which one of the values from Figure 6 is the relevant one. It seems reasonable to assume that the sublimation starts at surface atoms with the lowest coordination number. On one hand, the average binding energy of atoms with the coordination number of five is 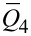 = (7.28 ± 0.23) × 10^−19^ J·atom^−1^ (Figure 6). On the other hand, it may be plausible that evaporation starts from the atom with the lowest binding energy. This is, as shown in Figure 6, *Q*_4,min_ = 6.792 × 10^−19^ J·atom^−1^. Certainly, this value is subject of some statistical scattering. However, these values should be, in any case, within the 3σ range of 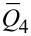 shown above, yielding a binding energy of a surface atom not smaller than Q_4,3σ_ = 3.95 × 10^5^ J·mol^−1^.

Assuming spherical clusters, both of the above approaches, Equation 1 and Equation 5, yield a surface energy formula in the form of γ = *C*/*a*. The constant *C* is given in Table 2. Consequently, the value of *C* from Equation 1 lies between the two values obtained for the application of the Kelvin equation for various binding energies. This fact, i.e., that atomistic (Equation 1) and continuum (Kelvin equation, Equation 5) give consistent predictions is an indication that both approaches are not too far from reality.

**Table 2:** Parameter *C* for calculating the surface energy γ. For the approach using the Kelvin equation, two parameters are given to characterize the 3σ scattering range for the exact value.

	*C* [J·cluster^−1^]
Equation 1	5.98 × 10^−18^
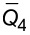 = 4.384 × 10^5^ J·mol^−1^	9.59 × 10^−18^
*Q*_4,3σ_ = 3.95 × 10^5^ J·mol^−1^	3.63 × 10^−18^

#### Determination of the cluster size

In order to apply Equation 1 or Equation 5 for calculating the surface energy, the surface area *a* of the cluster is needed. Generally, the area corresponding to the convex hull of all atomic positions is assumed as the surface of the cluster [30]. It can be determined using the software tool “qhull” [42]. However, using this approach, one neglects the fact that atoms themselves occupy space. Taking this fact into account, Medasani et al. [30,43] defined an “electron spill-out” parameter. In the case of silver, this led to an increase of the particle radius in the range of 0.05 to 0.08 nm. The influence of this correction is negligible for larger particles but becomes significant for small clusters, e.g., the Au_55_ cluster. The negligence of the actual size of the atoms leads to an increased surface energy for smaller particles.

**Figure 7:**
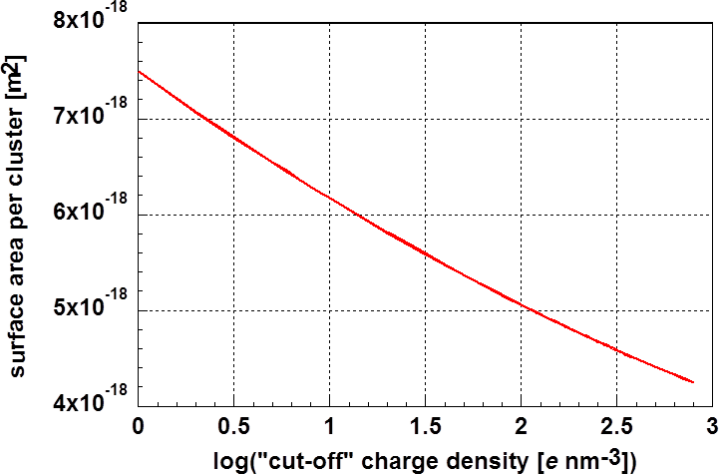
Surface area per Au_55_ cluster in the glassy phase as a function of the “cut-off” charge density.

Holec et al. [25] suggested resolving this situation by defining the cluster surface area as a convex hull of an electronic charge density higher than a certain constant “cut-off” value. The surface area *a* per cluster as a function of the “cut-off” charge density *q* is depicted in Figure 7.

**Figure 8:**
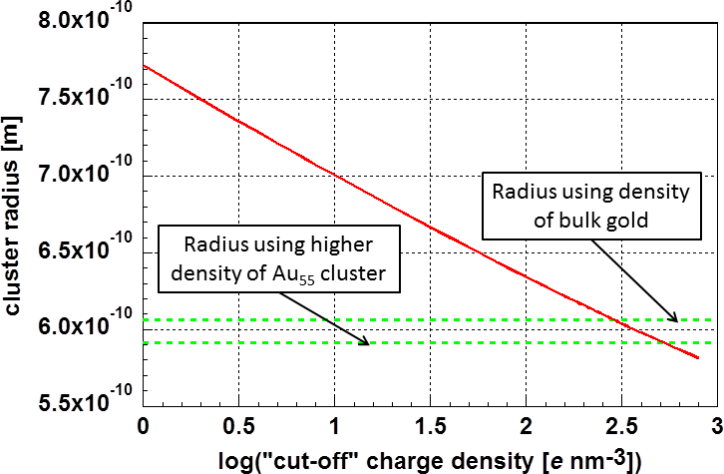
Dependency of the radius of the glassy Au_55_ cluster on the “cut-off” charge density. The calculated radii of the Au_55_ cluster assuming the mass density of bulk gold, as well as the calculated cluster radii considering the reduced atomic spacing (higher mass density) in the Au_55_ cluster, are marked.

Approximating the cluster with a sphere of equal surface allows for calculating the cluster radius *r* as a function of the “cut-off” charge density. This is displayed in Figure 8. Using the density of bulk gold (ρ = 1.933 × 10^4^ kg·m^−3^) as a starting value, a cluster radius of *r* = 6.06 × 10^−10^ m was calculated. Using for comparison the atomic spacing of bulk gold *d*_bulk_ = 2.855 × 10^−10^ m and *d*_Au55_ = 2.804 × 10^−10^ m, the reduced average atomic spacing determined for the Au_55_ cluster, a cluster radius of *r*_Au55_ = 5.91 × 10^−10^ m was determined. Both values are marked in Figure 8.

The dependence of the cluster radius on the logarithm of the cut-off charge density *q* is linear:

[6]
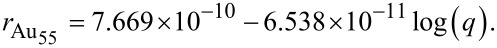


In Equation 6, the cluster radius *r*_Au55_ is given in meters and the cut-off charge density *q* in *e*·nm^−3^. This relation describes the dependency of the cluster radius on the cut-off charge density with a maximum deviation of less than 0.7%.

Using the surface-related energy quantities *C* given in Table 2 and the cluster radius of *r*_Au55_ = 5.91 × 10^−10^ m, it is finally possible to calculate the surface energy. The resulting values are summarized in Table 3.

**Table 3:** Surface energy γ of the glassy (most stable) Au_55_ cluster determined by two different methods. The confidence range for γ obtained by application of the Kelvin equation is 3σ.

calculation method	surface energy γ J·m^−2^

Medasani et al., Equation 1	1.36
Kelvin equation, Equation 5	1.51 ± 0.68

## Conclusion

The ab initio calculations performed within this study confirm the experimental results: (i) The most stable configuration of the Au_55_ cluster is not crystalline. This cluster is composed of two shells surrounding a central atom. With high probability this cluster is glassy, a “nanoglass” according to Gleiter [29]. The transition to the glassy structure is connected with an energy release equal to the melting enthalpy. Such a phenomenon is not observed for bulk materials, where the formation of the glassy structure is not connected with any energy release. (ii) The interatomic distance is smaller than in the fcc structure of bulk gold.

Furthermore, a shell structure should not be considered as a kind of ordering in the context of small nanoparticles. Even the random arrangement of gold atoms, used as starting condition for some of the calculations, shows such a shell structure (see Figure 2).

The non-equilibrium, crystalline cluster changes its structure during heating from crystalline at 0 K to amorphous, probably glassy, at 300 K. This phase transformation is suggested based on the analysis of the average coordination numbers and the total energies.

The data provided by our ab initio calculations allowed for the estimation of the surface energy by two entirely different approaches. Analyzing the values for the surface energy given in Table 3, one realizes that the more empirical approach of Medasani et al. [30], and the continuum thermodynamics-based approach employing the Kelvin equation, yield very close values of the surface energy. Furthermore, the total energy differences of various clusters are relatively small. The confidence interval of the results based on the Kelvin equation could be reduced by repeating the calculations, i.e., statistically evaluating several random clusters. In any case, this is the first determination of a surface energy giving a well-defined confidence interval.
